# Advanced Hydrogel-Based Strategies for Enhanced Bone and Cartilage Regeneration: A Comprehensive Review

**DOI:** 10.3390/gels9110885

**Published:** 2023-11-08

**Authors:** Diego De Leon-Oliva, Diego Liviu Boaru, Roque Emilio Perez-Exposito, Oscar Fraile-Martinez, Cielo García-Montero, Raul Diaz, Julia Bujan, Natalio García-Honduvilla, Laura Lopez-Gonzalez, Melchor Álvarez-Mon, Jose V. Saz, Basilio de la Torre, Miguel A. Ortega

**Affiliations:** 1 Department of Medicine and Medical Specialities, Faculty of Medicine and Health Sciences, University of Alcalá, 28801 Alcala de Henares, Spain; diegodleonoliva01@gmail.com (D.D.L.-O.); diego.boaru@edu.uah.es (D.L.B.); roquepe87@gmail.com (R.E.P.-E.); oscarfra.7@hotmail.com (O.F.-M.); cielo.gmontero@gmail.com (C.G.-M.); mjulia.bujan@uah.es (J.B.); natalio.garcia@uah.es (N.G.-H.); laura.lgonzalez@uah.es (L.L.-G.); mademons@gmail.com (M.Á.-M.); 2 Ramón y Cajal Institute of Sanitary Research (IRYCIS), 28034 Madrid, Spain; raul.diazp@uah.es (R.D.); josev.saz@uah.es (J.V.S.); bjtorre@gmail.com (B.d.l.T.); 3 Service of Traumatology of University Hospital Ramón y Cajal, 28034 Madrid, Spain; 4 Department of Surgery, Medical and Social Sciences, Faculty of Medicine and Health Sciences, University of Alcalá, 28801 Alcala de Henares, Spain; 5 Immune System Diseases-Rheumatology Service, Hospital Universitario Principe de Asturias, 28801 Alcala de Henares, Spain; 6 Department of Biomedicine and Biotechnology, Faculty of Medicine and Health Sciences, University of Alcalá, 28801 Alcala de Henares, Spain

**Keywords:** tissue engineering and regenerative medicine (TERM), advanced hydrogels, bone regeneration, extracellular matrix (ECM), scaffolds, stem cells (SCs)

## Abstract

Bone and cartilage tissue play multiple roles in the organism, including kinematic support, protection of organs, and hematopoiesis. Bone and, above all, cartilaginous tissues present an inherently limited capacity for self-regeneration. The increasing prevalence of disorders affecting these crucial tissues, such as bone fractures, bone metastases, osteoporosis, or osteoarthritis, underscores the urgent imperative to investigate therapeutic strategies capable of effectively addressing the challenges associated with their degeneration and damage. In this context, the emerging field of tissue engineering and regenerative medicine (TERM) has made important contributions through the development of advanced hydrogels. These crosslinked three-dimensional networks can retain substantial amounts of water, thus mimicking the natural extracellular matrix (ECM). Hydrogels exhibit exceptional biocompatibility, customizable mechanical properties, and the ability to encapsulate bioactive molecules and cells. In addition, they can be meticulously tailored to the specific needs of each patient, providing a promising alternative to conventional surgical procedures and reducing the risk of subsequent adverse reactions. However, some issues need to be addressed, such as lack of mechanical strength, inconsistent properties, and low-cell viability. This review describes the structure and regeneration of bone and cartilage tissue. Then, we present an overview of hydrogels, including their classification, synthesis, and biomedical applications. Following this, we review the most relevant and recent advanced hydrogels in TERM for bone and cartilage tissue regeneration.

## 1. Introduction

The skeleton is one of the largest organs in the human body, and it performs pivotal functions such as providing structural support for the body′s shape and facilitating movement. It also plays a crucial role in protecting vital organs and actively contributes to the overall homeostasis of the entire body by storing calcium and phosphate and harboring the bone marrow [1,2]. Articular cartilage represents a specialized type of connective tissue found in diarthrodial joints covering the surface of bones to allow almost frictionless joint movement and support load transfer, all while protecting the subchondral bone [3]. Bone and cartilage diseases, including bone fractures, tumors, osteoporosis, or osteoarthritis, significantly burden global healthcare systems and the quality of life for affected individuals. These conditions often lead to chronic pain, functional impairment, and reduced mobility, affecting millions of people worldwide [4,5,6]. There are instances in fracture healing where the process of bone regeneration faces challenges, particularly when fractures are too extensive to undergo natural regeneration. For example, up to 13% of tibia fractures are linked to delayed union or, in severe cases, fracture nonunion [7,8]. The conventional approach for treating patients whose bone treatments have been lengthy or unsuccessful is to use bone grafting, which ranks second among the most transplanted tissues in the United States, either as an allograft or an autograft [9]. However, there are issues with bone grafting. A more sustainable, less invasive, long-term healing strategy is consequently required to help treat fractures that have been damaged, and bone graft substitutes are being developed [10]. The following main approaches to bone restoration have been established, depending on the severity of the trauma: synthetic substitutes alone; scaffolds combined with active molecules; nanomedicine; cell-based combination products with different cells from various sources; biomimetic fibrous and nonfibrous substitutes; magnetic field and nano-scaffolds with stem cells; bioactive porous polymer/inorganic composite; and biomaterial-based 3D cell-printing substitutes [11,12,13,14,15,16,17]. The election of the different techniques is based on the healing potential and particular requirements of each case.

Langer and Vacanti defined tissue engineering in the early 1990s as “an interdisciplinary field which applies the principles of engineering and life sciences toward the development of biological substitutes that restore, maintain, or improve tissue function” [18]. In recent decades, there have been remarkable advances in the field of tissue engineering and regenerative medicine (TERM), with particular attention to the development of biomaterials-based strategies to facilitate tissue repair and regeneration [19,20]. Among these biomaterials, hydrogels have emerged as versatile and promising candidates for the promotion of the natural healing processes of bone and cartilage [21,22]. Their unique properties, including high water content, adjustable mechanical properties, and the ability to load bioactive molecules and cells, make hydrogels uniquely suited for TERM applications [23]. Hydrogel-based methods offer less invasive alternatives to conventional surgical techniques, reducing patient discomfort, shortening recovery times, and reducing complications associated with invasive procedures. In addition, these methods can be precisely tailored to the specific needs of each patient [24]. This personalized approach to regenerative therapies has the potential to deliver superior results while minimizing the likelihood of adverse reactions [25,26].

The main objectives of our article are to provide a comprehensive and current overview of advances in advanced hydrogels for bone and cartilage tissue regeneration. The review focuses especially on the recent advances in advanced hydrogels for bone and cartilage regeneration and the novelties of patient-specific treatments. This work arises from the need to address the increasingly prevalent disorders affecting bone and cartilage tissues and aims to bring together the specific contributions of advanced hydrogels in this context, thus contributing to the evolving field of TERM, moving toward personalized medicine. We collect the main knowledge on bone and cartilage structure and regeneration. Below, we present the state of the art of advanced hydrogels in TERM and explore their use in bone and cartilage tissue engineering. Through this review, we aim to illuminate the potentially transformative impact of hydrogel-based therapies in addressing unmet clinical demands associated with bone- and cartilage-related disorders.

## 2. Bone Biology

**Figure 1:**
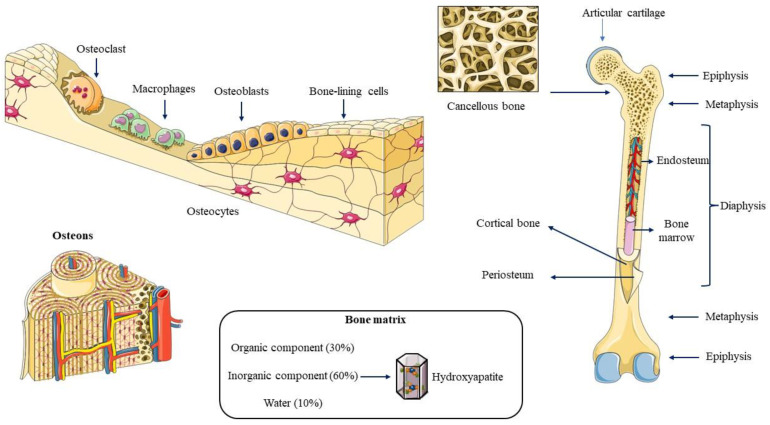
Bone anatomy and histology. A typical large bone exhibits a distinct structural organization, comprising epiphyses at the extremities housing bone marrow and cancellous bone, a central diaphysis characterized by a robust cortical bone layer, and the transitional metaphysis region. The bone tissue comprises various cell types alongside a mineralized extracellular matrix. The organic component, constituting approximately 30% of bone composition, primarily consists of collagen type I. In contrast, the inorganic component, representing around 60% of the bone’s composition, mainly comprises hydroxyapatite crystals.

Bone is a robust, multifunctional structure that plays a pivotal role in protecting vital organs, providing crucial kinematic support, regulating hematopoiesis, and preserving mineral balance. This dynamic tissue continuously remodels to maintain bone strength, mass, and the necessary calcium and phosphate mineral homeostasis, and importantly, it is the final step of bone regeneration [27]. It is formed by cells and a mineralized extracellular matrix (ECM) (Figure 1). Bone consists of five major cellular components: osteoprogenitor cells, osteoblasts, osteocytes, osteoclasts, and bone lining cells [2]. Osteoprogenitor cells, derived from mesenchymal stem cells (MSCs), are predominantly located in the bone marrow. These cells can differentiate into osteoblasts and maintain their osteoprogenitor potential in the adult bone system, making them crucial to the repair process [28,29]. Osteoblasts are recognized for their role in the formation of new bone via the secretion of the ECM [30,31]. Osteocytes are the most prevalent cells and are found in lacunae that are enclosed by a mineralized matrix, previously secreted as an osteoblast [32,33]. The osteoclasts are phagocytic multinucleated cells that have undergone terminal differentiation and are derived from the fusion of mononuclear cells of the hematopoietic stem cell (HSC) lineage under the effect of cytokines, such as receptor activator of nuclear factor κΒ ligand (RANKL) and macrophage colony-stimulating factor (M-CSF) [34,35,36]. Finally, the bone lining cells are quiescent flat-shaped osteoblasts that protect the surfaces of bones where neither bone growth nor bone resorption takes place [37]. 

The bone matrix is composed of organic compounds (30%), inorganic compounds (60%), and water (10%). The former is mainly composed of collagen type I (90%), and to a lesser extent collagen type V, and more than 30 proteins classified into four groups: proteoglycans, multiadhesive glycoproteins (osteonectin and podoplanin/E11), vitamin K-dependent proteins (osteocalcin and protein S) and growth factors (GFs), and cytokines (CKs) (IGF, TNFα, TGF-β, PDGF, BMP, IL-1, and IL-6) [38,39]. Hydroxyapatite (HA) crystals [Ca_10_(PO_4_)_6_(OH)_2_] are inorganic compounds, and their deposition is responsible for the mineralization of the bone matrix [40,41]. Research on the roles of bone matrix is essential for the development of hydrogel-based strategies for regenerative medicine.

**Figure 2:**
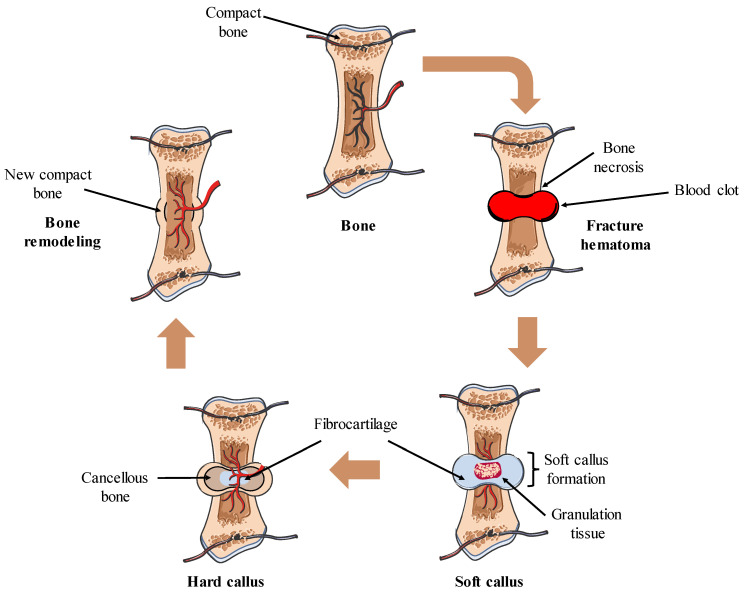
Bone healing process. The bone healing process comprises different phases. It begins with the formation of a hematoma and coagulation at the fracture site, followed by the recruitment of neutrophils and macrophages, together with the proliferation of fibroblasts and endothelial cells, leading to the formation of granulation tissue. Subsequently, a soft callus, characterized by a fibrocartilaginous matrix, connects the fractured bone ends. Osteoprogenitor cells of the periosteum differentiate into osteoblasts, initiating the formation of new bone around the soft callus. As fibrocartilage calcification occurs, new bone is deposited, culminating in the formation of a hard callus. Finally, bone remodeling occurs, restoring normal bone structure.

Bone regeneration is composed of a well-orchestrated sequence of biological events of bone induction and conduction that involve a variety of types of cells and intracellular and extracellular molecular-signaling pathways [42]. Contrary to other tissues, the majority of skeletal injuries, such as fractures, heal without the development of scar tissue. Instead, bone regenerates, with many of its pre-existing qualities restored, and finally becomes identical to the nearby uninjured bone [43,44]. The process of bone regeneration encompasses three sequential steps: the acute inflammatory response, bone repair, and remodeling (described in Figure 2) [45,46,47,48,49]. Surgical and non-surgical procedures play a crucial role in facilitating and expediting the bone-repair process by immobilizing the bones and bridging the gap between the two fractured ends [50,51]. With a greater understanding of bone biology at the molecular level, numerous new treatment approaches have been developed, and it is projected that there will be many more (or advancements to existing ones) in the years to come.

## 3. Cartilage Biology

Cartilage is an avascular, aneural, a-lymphatic connective tissue that is also present in the growth plates of children and adolescents. Humans have three different forms of cartilage: hyaline, fibrous, and elastic [52]. The chondrocytes, which produce and secrete the main elements of the extracellular matrix (EMC), are present in low density in all three types. The cartilage ECM has a special family of proteoglycans interwoven within a highly hydrated collagen fibrillar network to carry out the biomechanical responsibilities of giving structural support and resistance to deformation [53]. This matrix is produced and assembled by chondrocytes with the help of a large number of other non-collagenous proteins, proteoglycans, and glycoproteins. Each of the three forms of cartilage has varied abundance, distribution, and types of collagen and proteoglycans, which results in variations in appearance and biomechanical capabilities. The most prevalent type of cartilage in the human body is hyaline cartilage, which has a glassy appearance. It is located in the growth plates, ribs, nose, trachea, bronchi, and articulating surfaces of bones in synovial joints [54]. Articular cartilage is very similar to hyaline cartilage, except for the absence of perichondrium. It contains cartilage-specific collagen molecules that are crosslinked together in a copolymeric network, as well as proteoglycans and multiadhesive glycoproteins [55]. The limited regenerative capacity is due to the stability of cartilage-specific collagen molecules and the low activity of metalloproteinases.

Cartilage damage should ideally recover without leaving a scar. The smooth surface of the joint would be hampered by scar tissues inside the joint, and they would also have bad mechanical qualities [56]. Catabolic and anabolic damage responses in cartilage can be distinguished. The synthesis of agreement can degrade enzyme disintegrant and metalloproteinase with thrombospondin motifs 5 (ADAMTS-5), and the collagen matrix metalloproteinase 13 (MMP-13), which exclusively destroys type II collagen, is the catabolic activity that is most relevant to cartilage [57]. In reaction to injury, ADAMTS-5 is released quickly, but MMP-13 expression has a delayed response [58]. Instead, the anabolic activity involves the induction of chondroprotective genes like the transforming growth factor beta (TFG-β) family member activin A, hyaluronan-binding anti-inflammatory molecule, and tumor necrosis factor-inducible gene 6 protein (TSG-6). Uncertain factors may have contributed to the failure of cartilage regrowth [59,60]. Additional hints have been offered from the study of cartilage engineering: for instance, it is uncommon to see neocartilage integrating laterally with neighboring cartilage.

## 4. Hydrogels: Concept, Synthesis, and Biomedical Applications

Hydrogels are three-dimensional (3D) networks that are created by crosslinked polymers and can swell in aqueous solutions. These hydrogels possess unique properties such as biocompatibility, low toxicity, wide availability, viscoelasticity, and biodegradability, making them suitable for numerous biomedical applications [61,62,63,64]. These include TERM (reviewed in this paper), wound dressing, contact lenses, 3D cell cultures, drug delivery, antimicrobial resistance treatment, and biosensing. 

**Figure 3:**
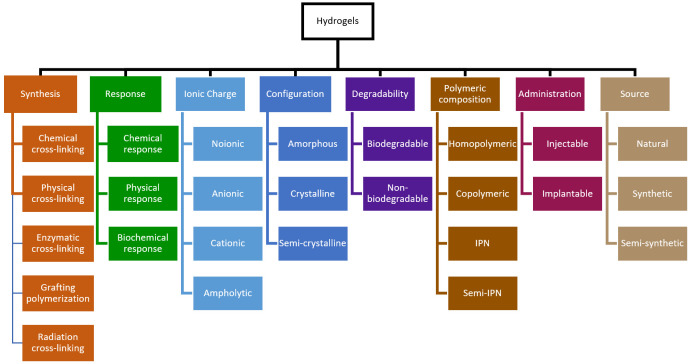
Classification of hydrogels according to different criteria.

Hydrogels can be classified according to different criteria (Figure 3), such as their source/composition, crosslinking, ionic charge, configuration, preparation, degradability, administration, and sensitivity to external stimuli [65]. Their diverse composition comprises natural polymers, synthetic polymers, or hybrid combinations of both. Natural polymers, from various biological origins, offer remarkable advantages, such as excellent biocompatibility, biodegradability, and non-toxicity [66,67,68]. Examples of natural polymers are chitosan, hyaluronic acid (HA), gelatin, alginate, and cellulose. In contrast, synthetic hydrogels contain synthetic polymers, making them ideal for tailoring and optimizing mechanical properties [69,70]. Some synthetic examples are polycaprolactone, poly (vinylpyrrolidone) (PVP), poly (lactic acid) (PLA), poly (ethylene glycol) (PEG), and poly (vinyl alcohol) (PVA).

Hydrogels are prepared through different procedures—chemical crosslinking, physical crosslinking, enzymatic crosslinking, grafting polymerization, and radiation crosslinking—which facilitate a broad spectrum of applications and fine-tuning of their characteristics to align with specific therapeutic and biomedical requirements [62,71,72]. Chemical crosslinking involves the establishment of covalent bonds that ensure long-term stability and durability [73,74]. For this purpose, crosslinking agents or the grafting of monomers onto the polymer backbone are used, and various chemical reactions can be employed, such as Schiff base reactions, photoelectric crosslinking, and crosslinking by chemical reactions of complementary groups [75,76,77]. In contrast, physical crosslinking relies on reversible interactions that provide flexibility and responsiveness to external stimuli [78]. These interactions include hydrogen bonding, the formation of amphiphilic grafting, crystallization, ionic interactions, maturation (heat-induced aggregation), and hydrophobic interactions, all of which contribute to the remarkable adaptability and multifunctionality of hydrogels in the biomedical field. These types of hydrogels are known for their ease of synthesis and lack of a crosslinking agent, which may have an impact on the reliability of loaded materials because of their toxicity [79,80]. Stress or changes in the physical environment can disrupt these interactions, allowing the hydrogel to revert to its polymer chains. Enzymatic crosslinking is a method in which an enzyme, such as tyrosinase, lysyl oxidase, or peroxidase, catalyzes the crosslinking within polymer chains [81,82,83]. They create in situ hydrogels characterized by exceptional biocompatibility. Grafting consists of the covalent attachment of a monomer to a polymeric host molecule or the polymerization of a monomer into a prefabricated polymeric skeleton [84,85,86,87]. This can be achieved using chemical or radiation-based methods, leading to the formation of functional hydrogels with specific properties. Radiation crosslinking uses sources such as electron beams, gamma radiation, or X-rays to generate free radicals in the polymer, resulting in crosslinking [88,89]. This method is preferred because of its ability to modify biopolymers without the need for chemical additives while ensuring biocompatibility and cost-effectiveness [90,91,92].

Hydrogels can also be classified into four main categories based on their polymeric composition: homopolymers, copolymers, interpenetrating polymer networks (IPNs), and semi-IPNs [62]. Homopolymer hydrogels are formed from a single monomer species, while copolymer hydrogels are derived from two or more monomer species and can be arranged as block, alternating, or random configurations along the chain of the polymer network [93,94]. Both present the same type of polymer. In contrast, semi-IPN hydrogels involve a polymeric network embedded within linear polymeric chains without crosslinking agents, whereas IPN hydrogels result from multiple polymeric networks crosslinked together using a crosslinking agent [95,96,97]. Semi-IPN and IPN hydrogels exhibit superior mechanical strength and swelling properties compared to homopolymeric and copolymeric hydrogels [98]. 

## 5. Hydrogels for TERM of Bone and Cartilage

Tissue engineering and regenerative medicine (TERM) is an emerging field that aims to achieve complete restoration of damaged tissues or organs. On the one hand, tissue engineering integrates the interplay of cells, scaffolds, and bioactive molecules to fabricate functional tissues. On the other hand, regenerative medicine encompasses a broader spectrum by synergizing tissue engineering with complementary strategies, such as cell therapy, gene therapy, and immunomodulation, all working in concert to promote tissue and organ regeneration [99]. In recent years, there have been significant advances in TERM, especially in the field of bone and articular cartilage regeneration [100,101].

In the field of TERM, hydrogels present ideal properties that make them suitable for bone regeneration and cartilage repair. Their ability to retain and release these therapeutic agents in a controlled manner facilitates tissue regeneration processes [102]. Moreover, the hydrophilic groups that retain water and the chemical crosslinker can also interact with the cells and molecules of the damaged tissue [103]. In addition, hydrogels offer the advantage of being customizable, allowing their physical and mechanical properties to be adjusted to those of the target tissue [102]. This fine tuning enhances their versatility and applicability in the field of tissue engineering and regenerative medicine (TERM). For example, hydrogels can be carefully designed to reproduce the characteristic stiffness and elasticity of specific tissues, facilitating seamless integration and optimal functionality in regenerative applications. In the following sections, we will provide an overview of some of the most prominent hydrogels used in TERM for bone and cartilage applications.

Hydrogels have diverse applications in the TERM of bone and cartilage defects. They play a crucial role in fracture healing by releasing bioactive molecules to accelerate the bone healing process and as bone graft substitutes that fill bone defects, eliminating the need for traditional bone grafting procedures [104,105,106,107]. Hydrogels act as supporting scaffolds, encapsulating cells, and growth factors to facilitate the formation of new bone tissue. In the treatment of osteoporosis, hydrogels are used to release drugs in a sustained manner and increase bone density [108,109,110,111,112]. In addition, they serve as lubricants in joints, offering relief to patients with joint diseases such as osteoarthritis [113,114,115,116,117]. In minimally invasive surgeries, injectable hydrogels conform to the irregular shapes of defects, providing structural support for bone and cartilage problems [118,119]. Finally, hydrogels are useful for local drug delivery, as they precisely target foci of bone infection with antibiotics, silver nanoparticles, or bacteriophages [120,121,122,123]. This variety of applications underscores the versatility and importance of hydrogels in meeting diverse clinical needs related to bone and cartilage regeneration. 

In the context of the TERM of bone and cartilage, physically crosslinked hydrogels are formed through reversible non-covalent interactions, offering high biocompatibility and suitability for applications requiring temporary support, flexibility, and rapid degradation [124,125]. Chemically crosslinked hydrogels, on the other hand, are created by covalent bonds, providing permanent and robust networks with increased mechanical strength. They are suitable for load-bearing applications due to their non-reversible nature and slower degradation rate, although concerns regarding residual chemicals must be taken into account [126,127]. The choice between these hydrogels in bone regeneration depends on specific clinical requirements, with physically crosslinked hydrogels being ideal for temporary and sensitive solutions and chemically crosslinked hydrogels being suitable for long-term stability and load-bearing applications [128].

**Figure 4:**
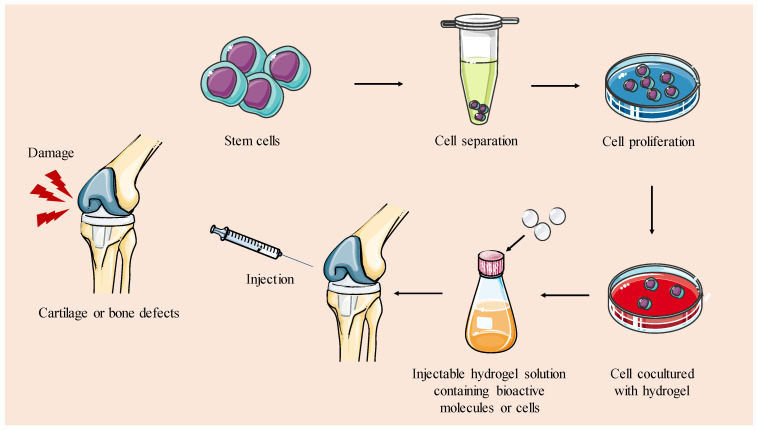
Schematic representation of strategies for creating injectable hydrogels intended for TERM applications in cartilage and bone. Injectable hydrogels are designed to solidify in situ via chemical reactions or the induction of physical factors to repair bone or cartilage defects.

Injectable hydrogels are a type of biomaterial that can be administered in liquid or gel form and then solidify or gel in situ at the desired site in the body [118,119]. Injectable scaffolds can be molded to fit bone/cartilage defects of any shape, which makes them a versatile option for various defect types and sizes and the most widely investigated technology in the field [129]. Injectable scaffolds also have the advantage of adhering well to the surrounding tissue, which promotes tissue integration. In addition, they minimize the need for aggressive surgical interventions, making them a more comfortable option for the patient [130]. These hydrogels are easily handled and can be loaded with cells, creating an environment conducive to cell survival and growth. This cell support facilitates specific cellular responses and guides the formation of new tissue (Figure 4). Lastly, they are suitable for drug delivery, allowing controlled release of bioactive molecules to enhance the regenerative process [131].

In the field of 3D bioprinting, bioinks based on naturally occurring hydrogels, such as (HA), gelatin, and fibrin, have demonstrated their ability to preserve crucial quality attributes of MSCs [132,133,134]. This preservation is attributed to the minimization of shear stress imparted by these bioinks, which not only provide structural support for cell proliferation but also facilitate osteogenic differentiation. Regarding cartilage tissue engineering, the main advantage of 3D bioprinting lies in its ability to hierarchically and spatially distribute 3D bioprinted cells, hydrogels, and active substances according to specific 3D requirements [135,136]. This approach generates an interconnected pore structure with substantial surface area, which facilitates cell adhesion, growth, intercellular communication, and gas and nutrient exchange [137]. These advantages represent a significant advance in promoting cartilage tissue regeneration compared to conventional solvent hydrogels. Bioinks are composed of conventional synthetic materials such as PVA, PAA, or nylon, charged with cells to construct allogeneic tissues and organs to avoid surgical invasive procedures, i.e., bone autografting [138]. Four-dimensional bioprinting goes beyond traditional three-dimensional bioprinting by incorporating the dimension of time as a critical factor in the printing process. In 4D bioprinting, hydrogels can respond to environmental stimuli, such as changes in temperature, pH, or the presence of specific molecules in a predetermined and programmable manner over time [139]. In the context of bone and cartilage TERM, shape memory implants adapt to evolving tissue needs, while stimuli-responsive scaffolds allow controlled cell differentiation through the release of growth factors [140]. These constructs mimic the behavior of natural tissue by altering its mechanical properties in response to mechanical loading. In addition, 4D-printed implants offer dynamic drug delivery to enhance regeneration and osteogenic maturation properties that promote osteoblast development [141]. Cartilage constructs adapt to changing joint conditions, promoting cartilage repair and joint function. Customized solutions can be achieved for each patient, ensuring that the regenerated tissue is precisely tailored to the anatomy and requirements of the patient.

### 5.1. Hydrogels in Bone Tissue Regeneration 

In bone tissue engineering (BTE), hydrogels need specific fundamental attributes: tissue and cell compatibility, along with osteoinductive and osteoconductive properties. Osteoinduction denotes the ability to stimulate osteogenesis, while osteoconduction refers to the capacity to facilitate bone growth on a surface or scaffold [142]. These essential attributes collectively contribute to the efficacy of the hydrogel in promoting bone regeneration and healing. The composition of hydrogels in bone tissue engineering (BTE) involves a hydrogel-inspired scaffold designed to reproduce the mechanical properties of the extracellular matrix (ECM) and facilitate bone remodeling [143]. Within this scaffold, cells with osteogenic potential, including several varieties of stem cells, are included along with bioactive molecules, mainly growth factors, and cytokines, aimed at recruiting immune and osteoprogenitor cells to the injured site [106]. This multifaceted structure is intended to recreate an environment conducive to bone regeneration [144]. The various techniques used to prepare hydrogels give rise to a wide range of options in terms of their structure. In the context of bone regeneration, the most commonly used forms are microbeads, nanogels, and hydrogel fibers [106].

In the field of bone regeneration, there have been significant advances in hydrogel research in recent years, accompanied by the emergence of numerous innovative strategies. In particular, drug delivery systems, especially bioactive molecules, and cell-loaded hydrogels, have shown promising results. Moreover, advanced hydrogels show multifunctional properties that improve the outcomes of bone tissue regeneration. Examples of hydrogels employed in the TERM of bone tissue are reported in Table 1.

#### 5.1.1. Bioactive Molecules-Loaded Hydrogels

Shekaran et al., designed a protease-degradable PEG synthetic hydrogel with a triple helical, α_2_β_1_ integrin-specific peptide (GFOGER) as a BMP-2 delivery vehicle [145]. This hydrogel showed susceptibility to matrix metalloproteinase (MMP) activity, resulting in a controlled and sustained release of BMP-2 at low doses in vivo. Consequently, it facilitated bone fracture regeneration by recruiting osteoprogenitor cells and bridging fracture sites in mice. Another example is SDF-1α/chitosan/carboxymeymethy-chitosan nanoparticles (NPs), which were prepared and added to thermosensitive chitosan/glycerol phosphate hydrogels [146]. These were injected into calvarial defects induced in rats. Bone regeneration was induced by the sustained release of SDF-1α in situ, which signals for the homing of host MSCs. Zhang et al., constructed an alginate hydrogel loaded with peptide nanofibers made of two ultrashort peptides for the self-assembly and the stimulation of the M2 phenotype [147]. Following this, the hydrogel was applied to treat bone defects in mice, and upon ultrasound stimulation, the nanofibers were released from the hydrogel. The M2 phenotype was induced in macrophages through the STAT6/PPAR-γ/SOCS3 signaling axis, leading to the inhibition of the production of reactive oxygen species (ROS) and secretion of BMP-2 and IGF-I that promote the osteogenic differentiation of bone marrow mesenchymal stem cells (BMSCs). Collectively, all this contributes to the bone regeneration of rats. Atsttrin is a derivative of progranulin, a secreted glycoprotein that binds to TNFα receptors and is involved in anti-inflammation, tissue repair, wound healing, and cartilage development [148]. Moradi et al., synthesized a chitosan/graphene oxide/hydroxyethyl cellulose/*β*-glycerol phosphate hydrogel loaded with Atsttrin [149]. Enhanced bone regeneration was observed using the injectable hydrogel, which facilitated the sustained release of Atsttrin, thereby promoting the establishment of a well-defined callus structure in a murine diabetic model. Wang and colleagues engineered a dynamic hydrogel mimicking the gel-like nature of a hematoma, crucially involved in the initial stages of bone repair, as we have reviewed above [150]. The gel was formulated by taking advantage of the reversible interaction between vancomycin and D-Ala-D-Ala dipeptide, effectively trapping and killing bacteria within the hematoma-like environment. In addition, the incorporation of an osteogenic peptide facilitated bone healing. This approach holds great promise for preventing infections in vulnerable bone fractures or cases of osteomyelitis. Lastly, hydrogels can also include plasmids. Cheng et al., loaded a glycol-based dendronized chitosan with G protein-coupled receptor kinase 2 interacting protein 1 (GIT1) plasmid [151]. MSCs were effectively transfected in vivo, which promotes bone repair and neovascularization around bone defects via the Notch signaling pathway. Calcium phosphate (CaP)-based products, including hydrogels, are very useful for bone tissue engineering [152,153]. Fatimi and colleagues developed an innovative cellulose-derived pH-sensitive hydrogel combined with biphasic calcium phosphate to create an injectable formulation aimed at enhancing bone regeneration. This injectable bone substitute exhibits osteoconductive characteristics and has demonstrated its ability to stimulate the formation of new bone tissue [154,155]. The addition of beta-tricalcium phosphate (β-TCP) particles in hydrogels significantly broadens the range of hydrogel stiffness and promotes osteogenic differentiation of human mesenchymal stem cells (hMSCs), with lower-stiffness composites showing the highest expression of alkaline phosphatase and gene markers associated with osteogenesis [156]. Svarca et al., added strontium ranelate and CaP nanoparticles to HA-based hydrogels for local osteoporosis treatment as a drug delivery system [157]. The incorporation of these components significantly affected hydrogel properties, such as swelling behavior, gel fraction, rheological properties, and microstructure, while strontium ranelate demonstrates a positive impact on cell viability, particularly within the concentration range of 0.05–0.2 μg/mL.

#### 5.1.2. Cells-Loaded Hydrogels

Stem cells from different sources are the most employed cells in hydrogel therapies due to their potential for proliferation and differentiation to regenerate the tissues. The treatment of osteoporosis with BMSCs in preclinical studies is effective [158]. Furthermore, hydrogels loaded with BMSCs have been used extensively in animal models of osteoporosis, with favorable results. A hydrogel composed of poloxamer 407 and HA was fortified with MnO_2_ to protect administered BMSCs from reactive oxygen species (ROS) accumulation in osteoporosis, thus effectively promoting bone regeneration [159]. In particular, this hydrogel induced an M2 phenotype of macrophages while reducing the expression of proinflammatory cytokines and the secretion of osteogenic factors such as TGF-β and PDGF. Human tonsil-derived mesenchymal stem cells (TMSCs) loaded on gelatin-hydroxyphenyl propionic acid hydrogel were delivered subcutaneously to the dorsal of ovariectomized mice [160]. It demonstrated a recovery of the femoral heads and serum osteocalcin and alkaline phosphatase. Interestingly, the mice also showed a reduction in visceral fat. 

Therefore, it can be deduced that in the development of hydrogels, attention must extend beyond just the degradation timeline. It is essential to take into account both the process of osteogenesis and osteodegradation and to carry out a thorough evaluation of constituents, their ratios, and fillers, which may prove invaluable in future research.

**Table 1:** Recent research on advanced hydrogels incorporating bioactive molecules and cells for bone tissue regeneration: composition, preparation, and the process of application and evaluation.

Polymer	Biological Factor	Mechanism of Gelation	Application	Year	References
PEG	BMP-2	Chemical crosslinking	Murine non-healing radial bone defect	2014	[145]
Chitosan/β-glycerol phosphate disodium salt	SDF-1α	Chemical crosslinking	Critical-sized calvarial defects in rats	2017	[146]
Calcium alginate	Ultrashort peptide nanofibers	Physical crosslinking	Rebuild osteogenic immune microenvironments	2024	[147]
Chitosan/graphene oxide/hydroxyethyl cellulose/β-glycerol phosphate	Atsttrin	Physical crosslinking	Bone regeneration in diabetic mice model	2023	[149]
Vancomycin/D-Ala-D-Ala/acrylamide	OGP	Physical crosslinking	Infected bone fracture	2023	[150]
Glycol-based dendronized chitosan	GIT1 plasmids	Physical crosslinking	Bone defects	2023	[151]
Hydroxypropylmethylcellulose	Biphasic calcium phosphate	Chemical crosslinking	New bone formation	2009/12	[154,155]
Agarose and agarose–collagen	β-TCP	Chemical crosslinking	Osteogenic differentiation of hMSCs	2018	[156]
HA	CaP NPs and strontium ranelate	Chemical crosslinking	Osteoporosis	2022	[157]
Poloxamer 407/HA	BMSCs	Chemical crosslinking	Osteoporosis	2023	[159]
Gelatin-hydroxyphenyl propionic acid	TMSCs	Chemical crosslinking	Postmenopausal osteoporosis	2018	[160]

### 5.2. Hydrogels in Cartilage Regeneration

TERM also takes advantage of the advantageous properties of hydrogels for articular cartilage regeneration, mainly their less invasive and easy application. These hydrogels, being swollen with water, offer a convenient means of effectively filling cartilage defects [60]. For these reasons, the most employed are injectable hydrogels, which homogenously distribute any shape before gelation. Within this, some common natural biomaterials are chitosan, collagen/gelatin, alginate, fibrin, elastin, heparin, chondroitin sulfate, and HA, while synthetic polymers include PEG, poly(L-glutamic acid), poly(vinyl alcohol), poly(propylene fumarate), *α*,*β*-poly-(*N*-hydroxyethyl)-DL-aspartamide, PEG-poly(*N*-isopropyl acrylamide), methoxy polyethylene glycol, and methoxy polyethylene glycol–poly(ε-caprolactone) [118]. The most common approaches found in the literature divide cell-free and cell-loaded hydrogels. Examples of hydrogels employed in TERM of cartilage tissue are reported in Table 2. 

#### 5.2.1. Cell-Free Hydrogels

Chitosan–gelatin hydrogels exhibit both durability and the ability to be finely adjusted in terms of their porosity and degradation rates [161]. In vitro, cell culture with cartilage cells of the human thyroid displays excellent adhesion, proliferation, and secretion of ECM. Lei et al., developed rapamycin-liposome-incorporating HA-based hydrogel microspheres to enhance joint lubrication, maintain cellular homeostasis, and mitigate the progression of osteoarthritis [162]. These microspheres formed self-renewable hydration layers, improved lubrication through a smooth rolling mechanism, and released rapamycin (autophagy activator) to target cartilage, ultimately offering effective lubrication and potential relief for friction-related conditions like osteoarthritis. Han et al., introduced injectable hydrogel microspheres (GelMA@DMA-MPCs) with enhanced lubrication and sustained drug release for the treatment of osteoarthritis (OA) [163]. These microspheres effectively improved lubrication, released diclofenac sodium (DS) for anti-inflammatory action, and demonstrated significant therapeutic effects in an OA rat model, offering a promising approach to the treatment of OA. Injectable marine collagen-based hydrogel effectively preserved the differentiated state of chondrocytes during in vitro culturing, a significant challenge in cartilage regeneration [164]. This biocompatible hydrogel formulation, capable of retaining cells without cytotoxic effects, allows for stiffness modulation and promotes chondrogenic gene expression (namely *Sox9*, *Col2A1*, and *Acan*). 

In other studies, an injectable double crosslinked hydrogel modified was developed with sodium alginate and gelatin, loaded with kartogenin (KGN) and TGF-β3 [165]. This cell-free hydrogel system attracted endogenous MSCs, induced chondrogenesis, and showed promise for cartilage repair in a one-step procedure. The study demonstrated the potential of the combination of KGN and TGF-β3 to promote MSC chondrogenesis for cartilage regeneration. Indeed, 4-aminobiphenyl (4-ABP) enzymatically derived from KGN significantly enhanced cartilage repair in a murine model of osteoarthritis. This improvement was achieved through the activation of the PI3K-Akt pathway, which in turn stimulates mesenchymal stem cell (MSC) proliferation and facilitates chondrogenic differentiation [166]. Similarly, Zhu et al., aimed to evaluate the efficacy of an integrated scaffold of 3D-printed decellularized cartilage extracellular matrix (ECM) and PEG diacrylate (PEGDA), in combination with the natural compound honokiol (Hon), to regenerate osteochondral defects [167]. Hon is a polyphenol extracted from Magnolia officinalis with pleiotropic properties, including anti-inflammation and anti-oxidant properties [168]. The research employed a controlled laboratory design using a rat model with cylindrical osteochondral defect in the trochlear groove of the femur. The results indicated that the PEGDA/ECM/Hon scaffold effectively reduced the release of proinflammatory cytokines from LPS-stimulated macrophages in vitro. In addition, the PEGDA/ECM/Hon group demonstrated superior results in terms of International Cartilage Repair Society (ICRS) score, micro-CT evaluation, and histological analysis, suggesting its potential as a promising hydrogel for the repair of osteochondral defects.

**Table 2:** Recent research on advanced hydrogels incorporating bioactive molecules and cells for cartilage regeneration: composition, preparation, and the process of application and evaluation.

Hydrogel	Core Material	Preparation	Application	Year	References
HA	Rapamycin-liposome microspheres	Physical crosslinking	Osteoarthritis	2022	[162]
Methacrylate gelatin hydrogel microspheres	Diclofenac sodium	Physical crosslinking	Osteoarthritis	2021	[163]
Marine collagen		Enzymatic crosslinking	Cartilage regeneration	2020	[164]
Sodium alginate and gelatin	KGN/TGF-β3	Double crosslinking	Cartilage regeneration	2020	[165,166]
PEGDA/ECM	Honokiol	Physical crosslinking	Osteochondral defect repair	2020	[167]
PEG-GelMA-HA	DPSCs	Physical crosslinking	Chondrogenic differentiation of DPSCs	2014	[169]
Carrageenan	MSCs	Physical crosslinking	3D bioprinting	2016	[170]
Chitosan glycerol phosphate/starch	ASCs	Physical crosslinking	Cartilage tissue engineering	2010	[171,172]
HSMSSA	Chondrocytes	Di-self-crosslinking	Cartilage repair fille	2020	[173]

#### 5.2.2. Cell-Loaded Hydrogels

Hydrogels provide a proper environment for loading cells, especially SCs. These protect the cells from high shear forces and improve the therapy. Nemeth et al., utilized ultraviolet-assisted capillary force lithography to create nanostructured scaffolds of composite PEG-GelMA-HA hydrogels, promoting the chondrogenic differentiation of dental pulp stem cells (DPSCs) [169]. DPSCs cultured on these nanopatterned scaffolds exhibited upregulation of chondrogenic gene markers and increased collagen type II deposition, indicating that nanotopography and HA cues are crucial for enhancing DPSC chondrogenesis. Thakur et al., developed 2D nanosilicate-reinforced κ-carrageenan hydrogels with shear-thinning properties, enhanced mechanical stiffness, and physiological stability for MSCs delivery [170]. Sa-Lima et al., investigated the development of injectable thermosensitive hydrogels based on chitosan glycerol phosphate (CGP) and starch for cartilage tissue engineering. These hydrogels showed minimal changes in transition temperature with increasing starch concentrations, making them suitable for minimally invasive applications [171]. The addition of starch improved the viscoelastic properties and degradation profile of the hydrogels. In a subsequent phase, the potential of the hydrogels to induce chondrocyte differentiation and cartilage matrix accumulation was evaluated, in particular with encapsulated adipose-derived stromal cells (ADSCs) [172]. The results indicated that chitosan-*β*-glycerophosphate-starch hydrogels, especially novel CST constructs, were promising for chondrogenic differentiation of ADSCs in cartilage tissue engineering using minimally invasive techniques. Yao et al., developed an injectable thiolated hyaluronic acid (HA-SH) and maleimided hyaluronic acid (HA-Mal) (HSMSSA) hydrogel, formed using thiol oxidation reactions and thiol/maleimide click chemistry, showed physicochemical properties affected by molecular weight and precursor concentration [173]. Although a single HSMSSA gel demonstrated moderate injectivity and promoted cartilage tissue formation, it lacked adhesion sites for efficient cell-cluster connections. Combining HSMSSA with bioactive collagen I in a self-crosslinking blend hydrogel improved degradation resistance, chondrocyte adhesion, and proliferation, together with an upregulation of gene expression levels associated with hyaline cartilage formation and proteoglycan secretion (collagen I, II and X, Sox 9, and aggrecan), making it a potential strategy for clinical cartilage repair fillers with expanded autologous chondrocytes. Overall, there is growing evidence supporting the potential usefulness of hydrogels for treating conditions affecting articular cartilage, such as osteoarthritis or rheumatoid arthritis.

## 6. Conclusions and Future Trends

In summary, advanced hydrogels hold great promise in the field of tissue engineering and regenerative medicine, especially for the regeneration of bone and cartilage tissues. Their exceptional physical and chemical characteristics, such as mechanical strength, water-retaining capacity, and ability to transport and deliver bioactive agents and cells, make them well suited to treat a variety of bone and cartilage defects. This is especially important for cartilage, whose natural regenerative capacity is limited. However, further research is crucial to optimize the efficacy of these hydrogels and accelerate their integration into clinical practices, where they can be used to treat a variety of conditions, such as bone fractures, infections, metastases, osteoporosis, or osteoarthritis.

Looking ahead, there are several critical findings and future trends to consider. Personalized therapies, facilitated by advances in 3D and 4D bioprinting, are expected to improve the precision of treatments. Stimuli-responsive hydrogels that react to physiological signals and combination therapies with hydrogels, growth factors, stem cells, and gene therapy could offer even more effective solutions. Regulatory approval, long-term safety and efficacy studies, and efforts to bridge the gap between research and commercialization will be essential steps. Patient education and global accessibility efforts will ensure that these cutting-edge hydrogel-based treatments reach a wide range of people in need, ultimately improving their quality of life.

## Data Availability

Not applicable.
